# Cognitive impairment across the schizophrenia spectrum: a comparative neuropsychological study

**DOI:** 10.3389/fpsyt.2025.1581756

**Published:** 2025-05-08

**Authors:** Xiao-Yan He, An-Pei Wei, Zhuo-Hui Huang, Fei Wang, Li Li Guo, Cai-Lan Hou

**Affiliations:** ^1^ The Affiliated Mental Health Center of Jiangnan University, Wuxi Central Rehabilitation Hospital, Wuxi, Jiangsu, China; ^2^ School of Humanities and Management Science, Wannan Medical College, Wuhu, Anhui, China; ^3^ Guang Dong Provincial People’s Hospital, Guangdong Academy of Medical Sciences, Guangdong Mental Health Center, Guangzhou, Guangdong, China

**Keywords:** cognitive functions, multi-episode chronic schizophrenia, first-episode psychosis, clinical high risk for psychosis, first-degree relatives of patients with schizophrenia

## Abstract

**Introduction:**

Cognitive impairment represents a core feature of schizophrenia spectrum disorders, predating psychosis onset and persisting throughout illness progression. This cross-sectional study systematically evaluates neurocognitive functioning across five critical populations: multi-episode chronic schizophrenia (MECS), first-episode psychosis (FEP), clinical high-risk (CHR) individuals, first-degree relatives (FDR), and healthy controls (HC).

**Methods:**

A comprehensive neuropsychological battery was administered to 366 participants: 40 MECS, 94 FEP, 54 CHR, 80 FDR, and 98 HC. Assessments included: Processing speed: Trail Making Test-A (TMT-A), Digit Symbol Coding Test (DST). Attention: Continuous Performance Test (CPT). Verbal memory: Hopkins Verbal Learning Test-Revised (HVLT-R).Visual memory: Brief Visuospatial Memory Test-Revised (BVMT-R). Executive function: Stroop Color and Word Test (SCWT). Group differences were analyzed using Analysis of Covariance (covariates: age, education) with Bonferroni correction, supplemented by multinomial logistic regression.

**Results:**

A stepwise gradient of impairment emerged across the schizophrenia spectrum (HC > FDR > CHR > FEP > MECS), with significant between-group differences (*p*<0.001) persisting after covariate adjustment. Key findings revealed: 1. MECS demonstrated cognitive deficits, showing significantly poorer verbal memory (HVLT-R:d=0.65:*p*<0.001) and executive function (Stroop words:d=0.56, p=0.003,Stroop color:d=0.60, *p*=0.006,Stroop words color:d=0.46, *p*=0.03) performance than FEP.2. CHR exhibited intermediate impairment: - Outperformed FEP in processing speed (TMT-A: d=0.45, p=0.009, DST: d=065, p=0.001), attention (CPT: d=0.42, *p*=0.03), and executive function (Stroop words color: d=0.39, *p*=0.03). - Underperformed FDR across all domains except visual memory (BVMT-R: d=0.16, *p*=0.92).3. FDR showed subtle but consistent deficits relative to HC (all tests *p*<0.05), with largest effects in visual memory (BVMT-R: d=0.99, <0.001).

**Discussion:**

These findings support a stratified continuum of cognitive impairment across schizophrenia progression. While chronic patients manifest generalized deficits, at-risk populations demonstrate distinct profiles: FDR display subclinical vulnerabilities, whereas CHR show intermediate impairment exceeding familial risk but preceding acute psychosis. The differential trajectory of verbal memory and executive function deficits from FEP to MECS suggests progressive deterioration in higher-order cognitive domains. These gradient patterns may inform stage-specific cognitive interventions across the schizophrenia spectrum.

## 1 Introduction

Schizophrenia, a chronic psychiatric disorder affecting approximately 1% of the global population, typically manifests in early adulthood with cognitive dysfunction constituting a core feature of its psychopathology (1, 2). Substantial evidence indicates that neurocognitive performance correlates significantly with disease progression, functional outcomes, and long-term prognosis in psychosis (3–5). Notably, cognitive deficits demonstrate particular clinical relevance through their association with attenuated responses to antipsychotic pharmacotherapy (6), while also serving as potential susceptibility markers and predictive indicators of psychosis risk. Emerging longitudinal data suggest that cognitive impairment frequently precedes the onset of overt psychotic symptoms (7, 8), highlighting its importance in early detection and intervention strategies.

Current research paradigms investigating pre-psychotic cognitive impairment primarily utilize two methodological approaches: studies of first-degree relatives of psychosis patients (FDR) and clinical high-risk (CHR) population investigations (9–11). Comparative analyses reveal that cognitive deficits in CHR individuals exhibit intermediate severity between those observed in established schizophrenia diagnoses and neurotypical controls (12). Crucially, neuropsychological performance metrics demonstrate predictive validity for psychosis transition in CHR cohorts, with converters showing marked deficits in intelligence quotient, verbal/visual memory, and processing speed relative to non-converting peers (13) patterns that may also portend poorer therapeutic responsiveness (14).

The FDR population similarly manifests moderate cognitive impairment, with twin studies revealing significant genetic correlations between cognitive performance measures and schizophrenia susceptibility (15). These findings position cognitive dysfunction as a critical endophenotype for elucidating the genetic architecture of schizophrenia. Importantly, both FDR and CHR populations present unique advantages for studying cognitive trajectory development, as their neuropsychological profiles remain unconfounded by antipsychotic exposure or chronic illness effects.

Despite these advances, critical inconsistencies persist in the literature regarding comparative cognitive impairment severity between CHR and FDR groups. While some studies report more pronounced deficits in CHR populations (16), others find comparable impairment levels across groups (17). Furthermore, the longitudinal course of cognitive functioning following first-episode psychosis (FEP) remains contentious, with evidence supporting both cognitive stability (90% of FEP patients maintaining stable trajectories over >2 years) (2, 18) and progressive decline models (19, 20).

To address these unresolved questions regarding cognitive trajectory evolution across schizophrenia spectrum disorders, the present study employs comprehensive neuropsychological assessment to comparatively analyze cognitive profiles in four critical populations: multiple-episode chronic schizophrenia (MECS) patients, first-episode psychosis (FEP) cases, clinical high-risk (CHR) individuals, and first-degree relatives (FDR) of psychosis patients. This study aims to compare cognitive function profiles across key stages of schizophrenia (MECS, FEP, CHR and FDR), and to identify stage-specific cognitive markers that may guide targeted interventions.

## 2 Methods

### 2.1 Study participants

This multicenter cross-sectional study recruited 366 participants from Guangdong Mental Health Center and Luo Ding Psychiatric Hospital, China, including five subgroups: 80 individuals with FDR [four participants were under the age of 18 (15–17 years old)], 94 FEP patients [eleven participants were under the age of 18 (15–17 years old)], 40 MECS patients, 54 individuals with CHR (five participants were under the age of 18 [15–17 years old)], and 98 healthy controls (HC) [two participants were under the age of 18 (15–17 years old)]. Detailed sample characteristics have been previously reported (21). All participants met the following inclusion criteria: (1) age 15–45 years; (2) the ability to understand the questionnaire and cooperate in completing all tests; (3) provision of written informed consent (parental/guardian consent for minors). Exclusion criteria encompassed: (1) neurological disorders (e.g., epilepsy, traumatic brain injury); (2) substance use disorders (ICD-10 criteria); (3) endocrine abnormalities (e.g., thyroid dysfunction); (4) severe systemic diseases; (5) intellectual disability affecting questionnaire comprehension. Diagnostic classifications followed established protocols. MECS: ICD-10 schizophrenia diagnosis with ≥2-year duration and multiple psychotic episodes. FEP: ICD-10 schizophrenia diagnosis with single episode and <1-year duration. CHR: Met Structured Interview for Prodromal Syndromes (SIPS) (22) criteria for one of the following specified conditions: Brief Intermittent Psychotic Symptoms (BIPS), Attenuated Psychotic Symptoms (APS) and Genetic Risk and Deterioration Syndrome (GRDS). Of the 54 clinical high-risk (CHR) individuals, 46 met diagnostic criteria for attenuated psychosis syndrome (APS), 6 for brief intermittent psychotic syndrome (BIPS), and 2 for genetic risk and deterioration syndrome (GRD). Stratified analyses were not performed due to the limited sample sizes in subgroup classifications. FDR: First-degree relatives of FEP patients not meeting CHR criteria. The HC group consisted of individuals who were confirmed by the Mini-International Neuropsychiatric Interview (MINI) (23) to have no psychiatric disorders meeting ICD-10 diagnostic criteria and had no family history of mental disorders.

Notably, CHR and FDR groups were medication-naïve. The study protocol (KY-Z-2022-052-02) received ethical approval from Guangdong Provincial People's Hospital Ethics Committee in accordance with the Declaration of Helsinki. Participants received ¥100 compensation.

### 2.2 Clinical assessments

Two psychiatrists with at least 3 years of research and clinical expertise created a form that was used to collect data on patient demographics, including sex, age, employment, marital status, and education status. The Chinese version of the Positive and Negative Syndrome Scale (PANSS) (24) was used to measure both negative and positive symptoms as well as overall psychopathology in the MECS, FDR, FEP, and CHR groups. The MINI was utilized to confirm specific diagnoses. Furthermore, each research participant's overall performance was measured via the Global Assessment of Functioning (GAF) (25), and depression symptoms were evaluated via the Montgomery-Asberg Depression Rating Scale (MADRS) (26).

### 2.3 Neurocognitive assessment

A section of the Chinese version Measurement and Treatment Research to Improve Cognition in Schizophrenia (MATRICS) Consensus Cognitive Battery (MCCB) was completed by the participants (27). The MCCB test was administered according to standardized guidelines provided in the test manual. In the previous Chinese psychiatric sample, test-retest reliabilities ranged from 0.73 to 0.94 (28). Testing for verbal learning was performed via the Hopkins Verbal Learning Test-Revised (HVLT-R), visual learning was executed via the Brief Visuospatial Memory Test-Revised (BVMT-R), processing speed was tested via the digit symbol coding test (DST) and Trail Making Test A (TMT-A), attention was tested via the continuous performance test (CPT), and executive function was examined via the Chinese version of the Stroop color and word test (SCWT). It is important to note that higher scores on tests other than the “TMT-A” test indicate better performance.

### 2.4 Statistical analyses

The measurement data are reported as s values, and the data were statistically analyzed via SPSS version 21.0 software. In the HC, MECS, FDR, FEP, and CHR groups, demographic characteristics, clinical features, and indicators of cognitive function were examined via the Pearson chi-square test or one-way analysis of variance (ANOVA). We also conducted multiple comparisons. When the variances were homogeneous, we used the LSD test, and when the variances were heterogeneous, we used Tamhane's T2 test. Analysis of covariance (ANCOVA) was utilized as a control variable in a one-way ANOVA to examine the variables that differed significantly across the five groups and accounted for demographic confounding. Cohen's d was used to compute effect sizes, which demonstrated the magnitude of the standardized mean difference in cognitive performance between the five groups. The associations between cognitive function and the SIPS, PANSS, GAF, and MADRS scores in the MECS, CHR, FDR, and FEP groups were examined via Spearman correlation analysis. The cognitive performance of the CHR group was examined via a dichotomous logistic regression analysis to gain an improved comprehension of its role. The independent variables were several items related to cognitive functioning (TMT-A, DST, CPT, HVLT-R, SCWT, and BVMT-R), and the dependent variables were the two-by-two subgroups of the four groups. Since the SIPS and PANSS were not assessed in all four groups, they were removed from the model analysis. The GAF total score and the MADRS total score are considered outcome variables. *p* < 0.05 was used to define statistical significance (two-tailed).

## 3 Results

### 3.1 Demographic and clinical characteristics of the participants

Significant intergroup differences emerged in sociodemographic variables (marital status, employment, and education level) across the five groups (**Table 1**). The results revealed significant differences among the MECS, FEP, CHR, and FDR groups, with a progressive decline in PANSS total scores (*F* = 391.5, *P* < 0.001), positive subscale scores (*F* = 186.15, *P* < 0.001), negative subscale scores (*F* =151.47, *P* < 0.001), and general psychopathology subscale scores (*F* =181.87, *P <*0.001). Specifically, symptom severity followed a descending gradient across groups: MECS > FEP > CHR > FDR. Notably, clinical measures demonstrated a characteristic gradient: GAF scores progressively increased (indicating better functioning),while MADRS scores sequentially decreased (indicating reduced depressive symptoms) across the severity spectrum from MECS → FEP → CHR → FDR → HC groups (**Table 2**).

**Table 1:** Sociodemographic of the multi-episode chronic schizophrenia, clinical-high risk, first-degree relatives, patients with first-episode schizophrenia and healthy control groups.

	MECS (n=40)		FEP (n=94)		CHR (n=54)		FDR (n=80)		HC (n=98)		Statistics		
	mean	SD			mean	SD	mean	SD	mean	SD	*F*	df	*P*
Age	27.43	4.97	26.24	7.57	28.24	7.46	27.21	7.28	28.44	7.72	1.27	4	0.28
	n	%	n	%	n	%	n	%	n	%	*χ^2^*	df	*P*
Female	15	37.5	34	36.2	27	50	38	47.5	49	50	5.59	4	0.23
Unmarried	22	55	65	69.1	23	42.6	44	55	49	50	11.94	4	**0.02**
Unemployed	25	60	71	75.5	21	38.9	29	36.2	20	20.4	67.48	4	**<0.001**
Educational level (years)											49.60	8	**<0.001**
<6	9	22.5	10	10.6	7	13.0	7	8.8	4	4.1			
6-12	29	72.5	75	79.8	42	77.8	57	71.2	54	55.1			
>12	2	5	9	9.6	5	9.2	16	20	40	40.8			

Bold values: *P<*0.05; CHR, clinical high risk for psychiatry; FDR, first-degree relatives of psychosis; FEP, first-episode schizophrenia; HC, healthy control; MECS, patients with multi-episode chronic schizophrenia.

**Table 2:** Clinical characteristics of the multi-episode chronic schizophrenia, clinical-high risk, first-degree relatives, patients with first-episode schizophrenia and healthy control groups.

	MECS (n=40)		FEP (n=94)		CHR (n=54)		FDR (n=80)		HC (n=98)		Statistics		
	mean	SD	mean	SD	mean	SD	mean	SD	mean	SD	*F*	df	*P*
PANSS Total	85.57	9.69	67.16	17.81	52.26	7.09	31.88	3.29	–	–	391.05	3	**<0.001**
PANSS Positive	21.40	4.61	17.19	6.12	10.17	2.75	7.20	0.56	–	–	186.15	3	**<0.001**
PANSS Negative	22.10	4.95	16.32	7.00	11.31	3.94	7.21	0.49	–	–	151.47	3	**<0.001**
PANSS General	42	5.47	33.82	8.30	30.78	5.93	17.49	3.16	–	–	181.87	3	**<0.001**
SIPS Total	–	–	–	–	18.75	7.39	0.68	1.95	0.83	1.50	443.77	2	**<0.001**
SIPS Positive	–	–	–	–	6.61	3.50	0.36	0.96	0.21	0.80	239.87	2	**<0.001**
SIPS Negative	–	–	–	–	6.07	3.50	0.24	0.89	0.30	0.71	209.64	2	**<0.001**
SIPS Disorganization	–	–	–	–	3.65	2.08	0.05	0.27	0.07	0.26	250.02	2	**<0.001**
SIPS General	–	–	–	–	2.17	2.47	0.05	0.27	0.23	0.60	53.31	2	**<0.001**
GAF Total	51.08	7.41	56.65	10.18	75.21	10.31	86.73	5.49	88.49	4.04	320.17	4	**<0.001**
MADRS Total	17.25	3.83	14.66	7.36	9.46	5.53	1.1	2.09	0.89	1.89	176.7	4	**<0.001**

Bold values: *P*<0.05; CHR, clinical high risk for psychiatry; FDR, first-degree relatives for psychosis; FEP, first-episode schizophrenia; HC, healthy control; PANSS, Positive and Negative Syndrome Scale; SIPS, Structured Interview for Psychosis-Risk Syndrome; GAF, Global Assessment Function; MADRS, Montgomery-Asberg Depression Rating Scale; MECS, patients with multi-episode chronic schizophrenia.

### 3.2 Comparison of study groups' cognitive performance

#### 3.2.1 MECS, FEP, CHR and FDR groups versus the HC group

ANCOVA revealed significant between-group differences between all five groups in the processing speed (TMT-A,*F*=42.48, *P*<0.001 and DST, *F*=75.44, *P*<0.001), verbal Learning (HVLT-R, *F*=60.69,*P*<0.001), visual learning (BVMT-R, *F*=35.66, *P*<0.001), attention (CPT, *F*=37.57,*P*<0.001), and executive function (Stroop Words *F*=52.11, *P*<0.001;Stroop Color, *F*=52.10, *P*<0.001;Stroop Color Words, *F*=46.00, *P*<0.001).

*Post hoc* analyses indicated that, compared to the HC group, the MECS group demonstrated significantly poorer performance across multiple cognitive domains: processing speed (TMT-A, *d* = 1.93, *P* < 0.001; DST, *d* = 2.25, *P* < 0.001),verbal learning (HVLT-R, *d* =2.68, *P <*0.001),visual learning (BVMT-R, *d* =1.90, *P* < 0.001),attention (CPT, *d* =1.63, *P <*0.001) and executive function (Stroop Words, *d* =2.28, *P <*0.001; Stroop Color, *d* = 2.45, *P <*0.001; Stroop Color-Words, d=2.07, *P <*0.001).Verbal learning emerged as the most severely impaired domain (*d* = 2.68).Similarly, the FEP group exhibited worse performance than HC in processing speed (TMT-A, *d* =1.44, *P <*0.001;DST,*d* = 2.13, *P <*0.001),verbal learning (HVLT-R,*d* =1.59, *P* < 0.001),visual learning (BVMT-R, *d* =1.39, *P* < 0.001),attention (CPT: *d* =1.65, *P <*0.001) and executive function (Stroop Words, *d* =1.50, *P* < 0.001; Stroop Color, *d* = 1.54, *P <*0.001;StroopColor-Words: *d* =1.50, *P <*0.001). Processing speed emerged as the most severely impaired domain (*d* = 2.13).

Compare to HC, the CHR group also displayed impairments in processing speed (TMT-A, *d* =1.26, *P <*0.001; DST, *d* =1.52, *P* < 0.001),verbal learning (HVLT-R, *d* =1.99, *P <*0.001),visual learning (BVMT-R, *d* =1.32, *P <*0.001),attention (CPT, *d* =1.09, *P* < 0.001) and executive function (Stroop Word, *d* =1.51, *P* < 0.001; Stroop Color, *d* =1.38, *P <*0.001; Stroop Color-Words, *d* = 1.17, *P <*0.001).Verbal learning emerged as the most severely impaired domain (*d* =1.99).

**Figure 1:**
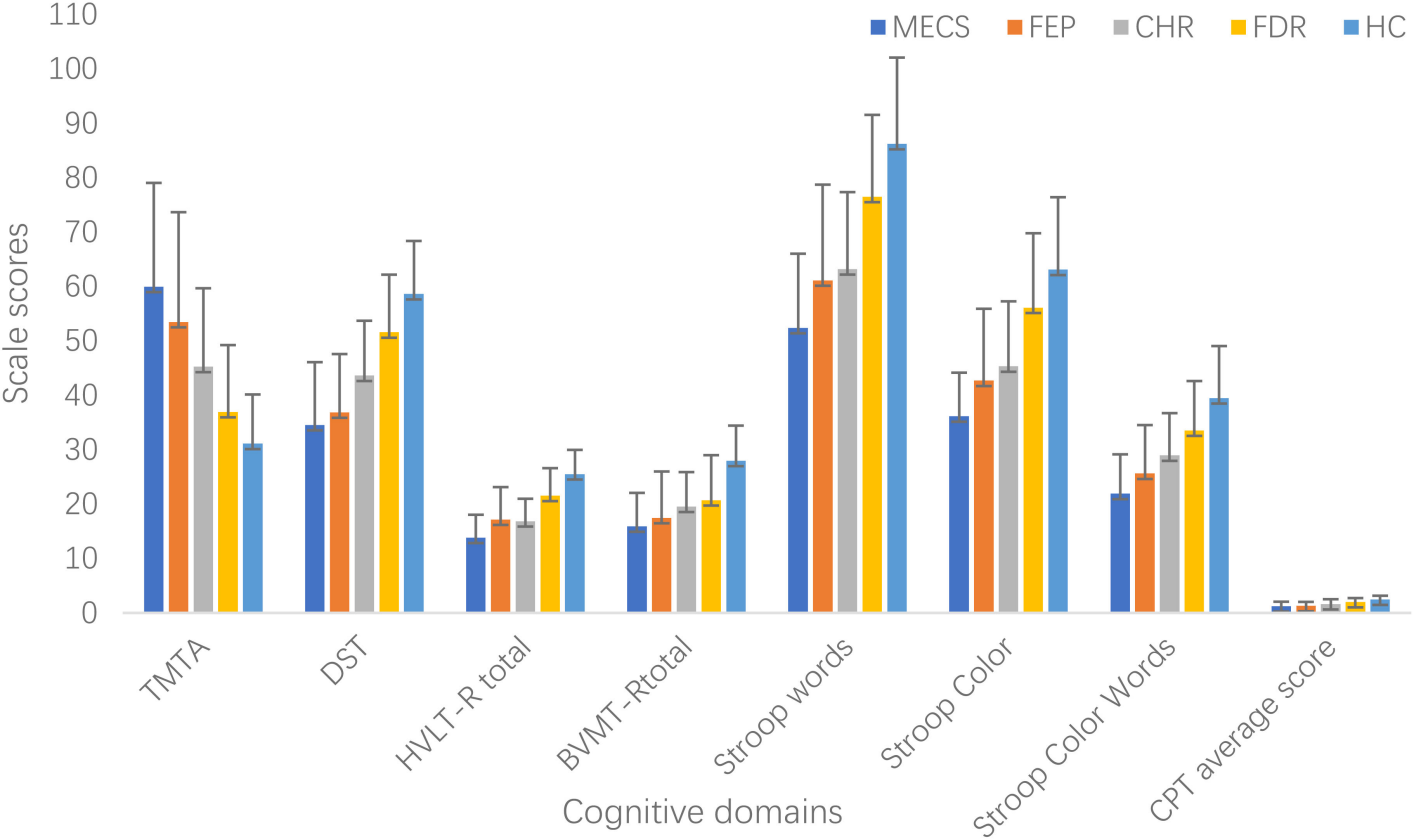
Cognitive function in different groups. BVMT-R, Brief Visuospatial Memory Test Revised; CHR, clinical high risk for psychiatry; CPT, Continuous Performance Test; DST, Digit Symbol Coding Test; FDR, first-degree relatives of psychosis; FEP, first-episode schizophrenia; HC, healthy control; HVLT-R, Hopkins Verbal Learning Test-Revised; MECS, patients with multi-episode chronic schizophrenia; TMT-A, Trail Making Test A.

Additionally, it is worth noting that compared to the HC group, the FDR group also exhibited impairments in processing speed (TMT-A, d=0.55, *P*=0.003; DST, d=0.69, *P*<0.001), verbal learning (HVLT-R, d=0.84, *P*<0.001), visual learning (BVMT-R, d=0.99, *P*<0.001), attention (CPT, d=0.64, *P*<0.001), and executive function (Stroop Words, d=0.63, *P*<0.001; Stroop Color, d=0.52, *P*<0.001; Stroop Executive Function, d=0.64, *P*<0.001). Visual learning is the most severely damaged area (d=1.99) (**Table 3**, **Figure 1**).

**Table 3:** Cognitive function of the multi-episode chronic schizophrenia, clinical-high risk, first-degree relatives, patients with first-episode schizophrenia and healthy control groups.

	MECS (n=40)		FEP (n=94)		CHR (n=54)		FDR (n=80)		HC (n=98)		Analysis of variance			Pairwise Comparison		
	mean	SD	mean	SD	mean	SD	mean	SD	mean	SD	*F*	df	*P*		*P*	ES
TMT-A (processing speed) ^#^	59.95	19.07	53.48	20.16	45.24	14.42	36.94	12.28	31.1	9.04	42.48	4	**<0.001**	MECS vs FEP	0.57	0.33
														MECS vs HC	**<0.001**	1.93
														FEP vs CHR	**0.03**	0.45
														FEP vs FDR	**<0.001**	0.97
														FEP VS HC	**<0.001**	1.44
														CHR vs FDR	**0.005**	0.63
														CHR vs HC	**<0.001**	1.26
														FDR vs HC	**0.003**	0.55
DST (Processing Speed) ^*^	34.55	11.53	36.85	10.72	43.61	10.08	51.56	10.6	58.6	9.75	75.44	4	**<0.001**	MECS vs FEP	0.24	0.21
														MECS vs HC	**<0.001**	2.25
														FEP vs CHR	**0.001**	0.65
														FEP vs FDR	**<0.001**	1.38
														FEP VS HC	**<0.001**	2.13
														CHR vs FDR	**<0.001**	0.77
														CHR vs HC	**<0.001**	1.52
														FDR vs HC	**<0.001**	0.69
HVLT-R T1^#^	3.63	1.41	4.26	1.88	4.2	1.52	5.28	1.86	6.52	1.8	32.03	4	**<0.001**	MECS vs FEP	0.30	0.38
														MECS vs HC	**<0.001**	1.78
														FEP vs CHR	0.87	0.03
														FEP vs FDR	**<0.001**	0.55
														FEP vs HC	**<0.001**	1.3
														CHR vs FDR	**0.001**	0.62
														CHR vs HC	**<0.001**	1.36
														FDR vs HC	**<0.001**	0.68
HVLT-R T2 ^#^	4.73	1.53	5.97	2.17	5.89	1.55	7.51	1.88	8.76	1.77	50.53	4	**<0.001**	MECS vs FEP	**0.003**	0.66
														MECS vs HC	**<0.001**	2.44
														FEP vs CHR	1	0.04
														FEPvs FDR	**<0.001**	0.75
														FEPvs HC	**<0.001**	1.41
														CHR vs FDR	**<0.001**	0.92
														CHR vs HC	**<0.001**	1.69
														FDR vs HC	**<0.001**	0.69
HVLT-R T3^#^	5.48	1.81	6.95	2.53	6.78	1.93	8.74	1.95	10.21	1.59	58.10	4	**<0.001**	MECS vs FEP	**0.002**	0.67
														MECS vs HC	**<0.001**	2.77
														FEP vs CHR	0.99	0.07
														FEP vs FDR	**<0.001**	0.78
														FEP vs HC	**<0.001**	1.55
														CHR vs FDR	**<0.001**	1.01
														CHR vs HC	**<0.001**	2
														FDR vs HC	**<0.001**	0.85
HVLT-R total(**v**erbal learning)^#^	13.83	4.26	17.17	5.95	16.87	4.11	21.54	5.06	25.51	4.45	60.69	4	**<0.001**	MECS vs FEP	**<0.001**	0.65
														MECS vs HC	**<0.001**	2.68
														FEP vs CHR	1	0.06
														FEP vs FDR	**<0.001**	0.79
														FEP vs HC	**<0.001**	1.59
														CHR vs FDR	**<0.001**	0.99
														CHR vs HC	**<0.001**	1.99
														FDR vs HC	**<0.001**	0.84
BVMT-R 1^#^	3.93	1.89	3.73	2.54	4.33	2.2	4.64	2.76	7.02	2.8	26.47	4	**<0.001**	MECS vs FEP	1	0.09
														MECS vs HC	**<0.001**	1.29
														FEP vs CHR	0.18	0.25
														FEP vs FDR	**0.02**	0.34
														FEP vs HC	**<0.001**	1.23
														CHR vs FDR	0.51	0.12
														CHR vs HC	**<0.001**	1.03
														FDR vs HC	**<0.001**	0.86
BVMT-R 2^#^	5.38	2.36	6.22	3.22	6.87	2.55	7.21	3.16	9.91	2.57	30.60	4	**<0.001**	MECS vs FEP	0.12	0.3
														MECS vs HC	**<0.001**	1.84
														FEP vs CHR	0.69	0.22
														FEP vs FDR	0.23	0.31
														FEP vs HC	**<0.001**	1.27
														CHR vs FDR	0.98	0.12
														CHRVS HC	**<0.001**	1.19
														FDR vs HC	**<0.001**	0.95
BVMT-R 3^#^	6.53	2.71	7.51	3.39	8.33	2.69	8.79	3.2	11.02	1.86	29.46	4	**<0.001**	MECS vs FEP	0.06	0.09
														MECS vs HC	**<0.001**	1.93
														FEP vs CHR	0.69	0.22
														FEP vs FDR	0.23	0.31
														FEP vs HC	**<0.001**	1.29
														CHR vs FDR	0.98	0.12
														CHR vs HC	**<0.001**	1.19
														FDR vs HC	**<0.001**	0.95
BVMT-R total(visual learning)^#^	15.90	6.16	17.47	8.52	19.54	6.34	20.73	8.26	27.97	6.45	35.66	4	**<0.001**	MECS vs FEP	0.26	0.21
														MECS vs HC	**<0.001**	1.90
														FEP vs CHR	0.45	0.27
														FEP vs FDR	0.07	0.39
														FEP vs HC	**<0.001**	1.39
														CHR vs FDR	0.92	0.16
														CHRVS HC	**<0.001**	1.32
														FDR vs HC	**<0.001**	0.99
Stroop Words (Executive functioning)*	52.38	13.63	61.12	17.58	63.17	14.16	76.48	15.04	86.19	15.86	52.11	4	**<0.001**	MECS vs FEP	**0.003**	0.56
														MECS vs HC	**<0.001**	2.28
														FEP vs CHR	0.45	0.13
														FEP vs FDR	**<0.001**	0.93
														FEP vs HC	**<0.001**	1.5
														CHR vs FDR	**<0.001**	0.91
														CHR vs HC	**<0.001**	1.51
														FDR vs HC	**<0.001**	0.63
Stroop Color (Executive functioning)*	36.13	8.02	42.69	13.2	45.31	11.95	56.11	13.66	63.08	13.31	52.10	4	**<0.001**	MECS vs FEP	**0.006**	0.60
														MECS vs HC	**<0.001**	2.45
														FEP vs CHR	0.24	0.21
														FEP vs FDR	**<0.001**	1
														FEP vs HC	**<0.001**	1.54
														CHR vs FDR	**<0.001**	0.83
														CHRVS HC	**<0.001**	1.38
														FDR vs HC	**<0.001**	0.52
Stroop Color Words (Executive functioning)*	21.9	7.24	25.61	8.91	28.94	7.77	33.53	9.08	39.47	9.57	46.00	4	**<0.001**	MECS vs FEP	**0.03**	0.46
														MECS vs HC	**<0.001**	2.07
														FEP vs CHR	**0.03**	0.39
														FEP vs FDR	**<0.001**	0.88
														FEP vs HC	**<0.001**	1.50
														CHR vs FDR	**0.004**	0.54
														CHRVS HC	**<0.001**	1.17
														FDR vs HC	**<0.001**	0.64
CPT2D#	1.90	1.19	1.95	0.99	2.27	1.17	2.73	0.85	3.25	0.82	27.71	4	**<0.001**	MECS vs FEP	1	0.05
														MECS vs HC	**<0.001**	1.32
														FEP vs CHR	0.43	0.30
														FEP vs FDR	**<0.001**	0.84
														FEP vs HC	**<0.001**	1.43
														CHR vs FDR	0.08	0.46
														CHRVS HC	**<0.001**	1.02
														FDR vs HC	**<0.001**	0.62
CPT3D*	1.24	0.91	1.32	0.84	1.66	1.05	2.14	0.91	2.57	0.82	31.43	4	**<0.001**	MECS vs FEP	0.6	0.09
														MECS vs HC	**<0.001**	1.54
														FEP vs CHR	**0.03**	0.37
														FEP vs FDR	**<0.001**	0.95
														FEP vs HC	**<0.001**	1.51
														CHR vs FDR	**0.002**	0.50
														CHRVS HC	**<0.001**	1
														FDR vs HC	**0.001**	0.50
CPT4D*	0.63	0.61	0.67	0.61	1	0.73	1.21	0.77	1.58	0.82	24.18	4	**<0.001**	MECS vs FEP	0.77	0.07
														MECS vs HC	**<0.001**	1.31
														FEP vs CHR	**0.008**	0.50
														FEP vs FDR	**<0.001**	0.79
														FEP vs HC	**<0.001**	1.26
														CHR vs FDR	0.11	0.28
														CHRVS HC	**<0.001**	0.74
														FDR vs HC	**0.001**	0.46
CPT average score (attention)*	1.26	0.79	1.31	0.72	1.64	0.88	2.02	0.71	2.47	0.69	37.57	4	**<0.001**	MECS vs FEP	0.68	0.07
														MECS vs HC	**<0.001**	1.63
														FEP vs CHR	**0.009**	0.42
														FEP vs FDR	**<0.001**	0.99
														FEP vs HC	**<0.001**	1.65
														CHR vs FDR	**0.003**	0.49
														CHRVS HC	**<0.001**	1.09
														FDR vs HC	**<0.001**	0.64

Bold values: *P<*0.05; **^#^** use Tamhane'sT2 for multiple comparisons when equal variances not assumed; *use LSD for multiple comparisons when equal variances assumed; BVMT-R, Brief Visuospatial Memory Test Revised; CHR, clinical high risk for psychiatry; CPT, Continuous Performance Test; DST, Digit Symbol Coding Test; FDR, first-degree relatives of psychosis; FEP, first-episode schizophrenia; HC, healthy control; HVLT-R, Hopkins Verbal Learning Test-Revised; MECS, patients with multi-episode chronic schizophrenia; TMT-A, Trail Making Test A.

#### 3.2.2 MECS group versus FEP group

**Figure 2:**
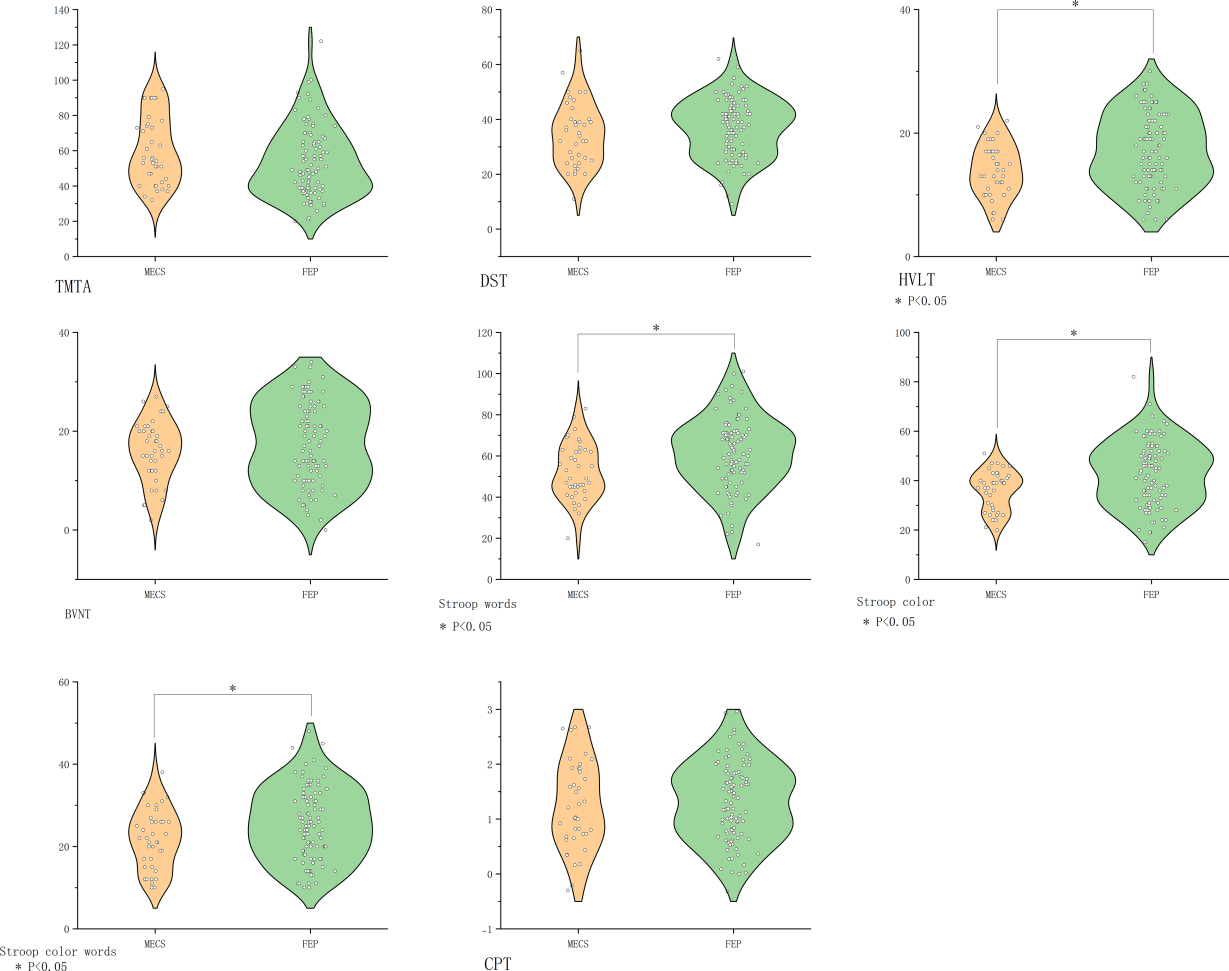
Comparison of cognitive function in the MECS group vs. the FEP group. BVMT-R, Brief Visuospatial Memory Test Revised; CHR, clinical high risk for psychiatry; CPT, Continuous Performance Test; DST, Digit Symbol Coding Test; FDR, first-degree relatives of psychosis; FEP, first-episode schizophrenia; HC, healthy control; HVLT-R, Hopkins Verbal Learning Test-Revised; MECS, patients with multi-episode chronic schizophrenia; TMT-A, Trail Making Test A. The symbol “*” means that the difference between the two groups after t-test is statistically significant with a p-value less than 0.05.

MECS patients exhibited pronounced deficits in verbal learning (HVLT-R, *P*<0.001) and executive function (SCWT subtests: Words *P*=0.003, Color *P*=0.006, Color-Word *P*=0.03). No significant differences emerged in other domains (**Figure 2**).

#### 3.2.3 FES group versus. CHR group

**Figure 3:**
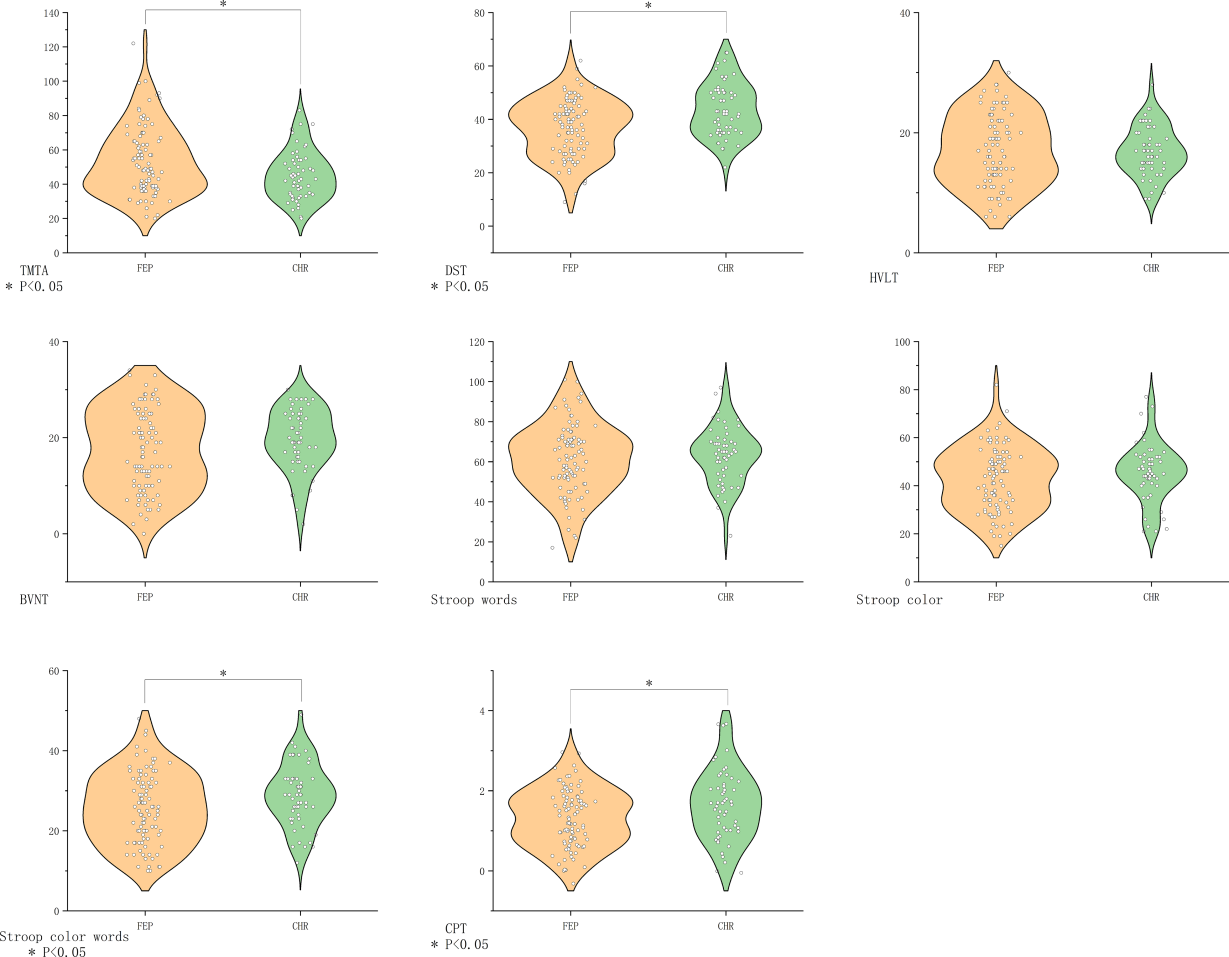
Comparison of cognitive function in the FEP group vs. the CHR group. BVMT-R, Brief Visuospatial Memory Test Revised; CHR, clinical high risk for psychiatry; CPT, Continuous Performance Test; DST, Digit Symbol Coding Test; FDR, first-degree relatives of psychosis; FEP, first-episode schizophrenia; HC, healthy control; HVLT-R, Hopkins Verbal Learning Test-Revised; MECS, patients with multi-episode chronic schizophrenia; TMT-A, Trail Making Test A. The symbol “*” means that the difference between the two groups after t-test is statistically significant with a p-value less than 0.05.

Compared with the CHR group, the FEP group showed greater impairment in processing speed (DST *P*=0.001, TMT-A *P*=0.03), attention (CPT *P*=0.009), and executive function (SCWT Color-Word *P*=0.03). Verbal/visual memory and other executive measures did not differ significantly (**Figure 3**).

#### 3.2.4 FEP group versus. FDR group

**Figure 4:**
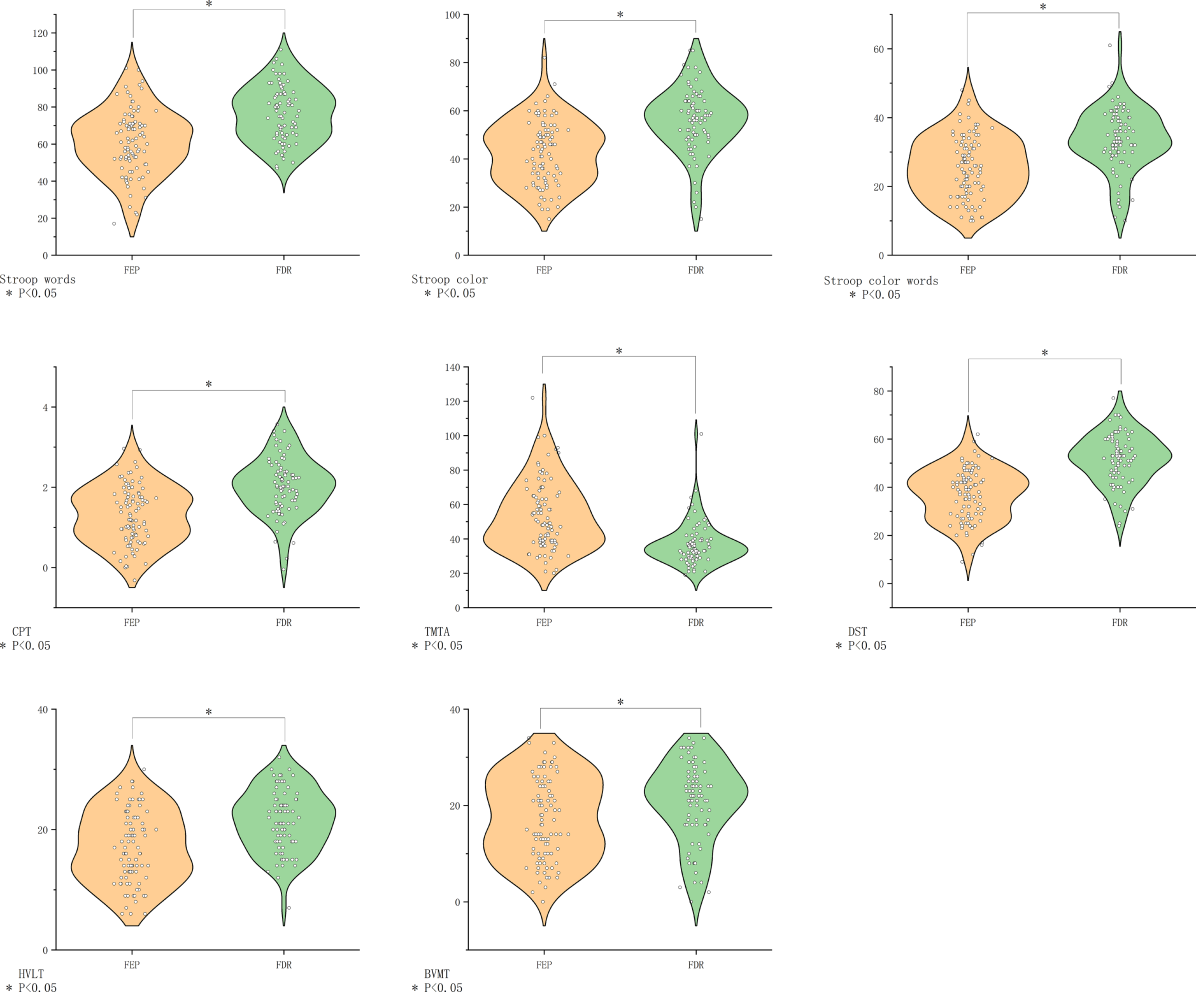
Comparison of cognitive function in the FEP group vs. the FDR group. BVMT-R, Brief Visuospatial Memory Test Revised; CHR, clinical high risk for psychiatry; CPT, Continuous Performance Test; DST, Digit Symbol Coding Test; FDR, first-degree relatives of psychosis; FEP, first-episode schizophrenia; HC, healthy control; HVLT-R, Hopkins Verbal Learning Test-Revised; MECS, patients with multi-episode chronic schizophrenia; TMT-A, Trail Making Test A. The symbol “*” means that the difference between the two groups after t-test is statistically significant with a p-value less than 0.05.

FEP patients demonstrated broad cognitive deficits compared to FDR (all *P*<0.05), except in visual memory (BVMT-R, *P*>0.05) (**Figure 4**).

#### 3.2.5 CHR group versus FDR group

**Figure 5:**
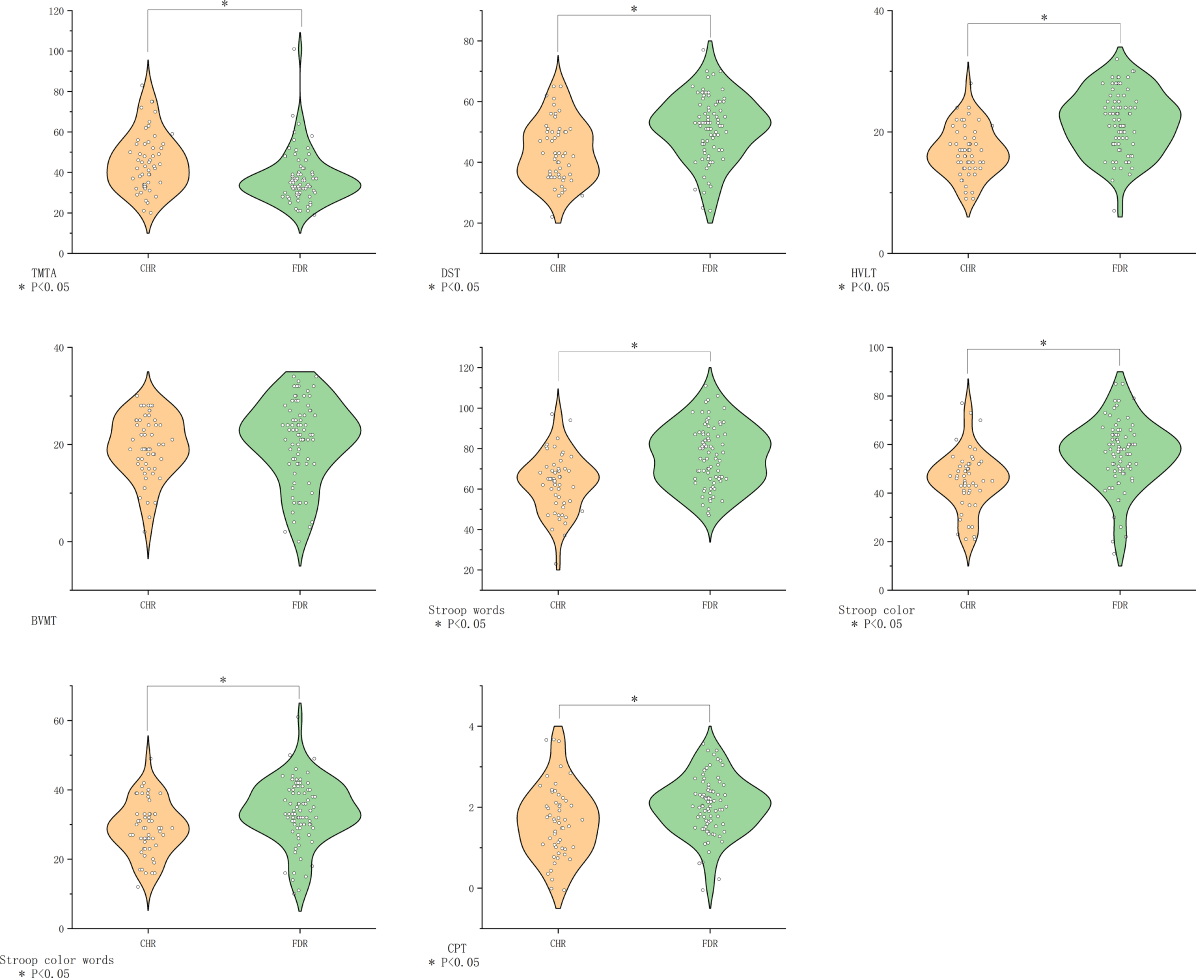
Comparison of cognitive function in the CHR group vs. the FDR group. BVMT-R, Brief Visuospatial Memory Test Revised; CHR, clinical high risk for psychiatry; CPT, Continuous Performance Test; DST, Digit Symbol Coding Test; FDR, first-degree relatives of psychosis; FEP, first-episode schizophrenia; HC, healthy control; HVLT-R, Hopkins Verbal Learning Test-Revised; MECS, patients with multi-episode chronic schizophrenia; TMT-A, Trail Making Test A. The symbol “*” means that the difference between the two groups after t-test is statistically significant with a p-value less than 0.05.

CHR individuals showed inferior performance across all domains except visual memory (BVMT-R), with maximal divergence in verbal learning (HVLT-R, *P*=0.001, d=0.99) (**Figure 5**).

### 3.3 Cognitive-clinical correlations

In the MECS group, the attention (average CPT score) was positively correlated with the general function (GAF score).

In the FEP group, the processing speed, attention, verbal memory and executive function (DST, CPT, HVLT-R, Stroop words, Stroop color, Stroop word color and BVMT-R total scores) were negatively associated with the psychiatric symptom [PANSS scores (all *p* < 0.05)]. The verbal memory and executive function (HVLT-R, Stroop word and Stroop color scores) were negatively associated with depression symptoms (the MADRS). Processing speed (TMT-A scores) and positively correlated with the depression symptoms and psychiatric symptom (MADRS and PANSS total scores).

Within the CHR group, the attention and processing speed (average CPT scores and the DST scores) were positively associated with general function [the scores of the GAF scale (*p* < 0.05)], and the processing speed (TMT-A scores) were negatively associated with general function [the scores of the GAF scale (*p* < 0.05)].

In the FDR group, visual memory and attention (BVMT-R total scores, and CPT average scores) were positively related to the general function [GAF scale score (*p* < 0.05)]. Conversely, the executive function, visual memory and attention (Stroop color, BVMT-R total scores and CPT average scores) were negatively correlated with depression symptoms [the total score on the MADRS (*p* < 0.05)] (**Table 4**).

**Table 4:** Correlation between cognitive function and scores of SIPS, PANSS, GAF, and MADRS.

	MECS			FEP			CHR			FDR		
	PANSS	GAF	MADRS	PANSS	GAF	MADRS	SIPS	GAF	MADRS	SIPS	GAF	MADRS
TMT-A	-0.14	-0.17	-0.10	0.36**	-0.08	0.21*	0.09	-0.45**	0.004	0.06	0.06	-0.22
DST	-0.05	0.27	0.07	-0.15	0.09	-0.06	0.06	0.38**	-0.27	-0.12	0.28*	-0.21
HVLT-R total	0.03	0.001	-0.18	-0.49**	0.07	-0.25*	-0.27	0.20	-0.14	0.04	0.11	-0.16
BVMT-R total	0.01	0004	-0.07	-0.28**	0.11	-0.17	-0.16	0.19	-0.08	-0.06	0.31**	-0.26*
Stroop words	0.10	0.13	-0.04	-0.44**	0.07	-0.22*	-0.08	0.22	-0.02	0.17	0.19	-0.08
Stroop color	0.03	0.09	-0.11	-0.47**	0.06	-0.23*	-0.15	0.19	-0.07	0.13	0.21	-0.25*
Stroop color words	-0.19	0.07	-0.03	-0.44**	0.09	-0.14	-0.22	0.15	-0.04	-0.02	0.11	-0.17
CPT average score	-0.19	0.32*	-0.15	-0.21*	0.27**	0.03	-0.11	0.27*	-0.12	-0.14	0.43**	-0.37**

*Correlation is significant at the 0.05 level (2-tailed), **Correlation is significant at the 0.01 level (2-tailed). BVMT-R, Brief Visuospatial Memory Test Revised; CHR, clinical high risk for psychiatry; CPT, Continuous Performance Test; DST, Digit Symbol Coding Test; FDR, first-degree relatives of psychosis; FEP, first-episode schizophrenia; HC, healthy control; HVLT-R, Hopkins Verbal Learning Test-Revised; MECS, patients with multi-episode chronic schizophrenia; PANSS, Positive and Negative Syndrome Scale; SIPS, Structured Interview for Psychosis-Risk Syndrome; TMT-A, Trail Making Test A.

### 3.4 Cognitive risk factors distinguishing FEP and CHR groups: results of binary logistic regression analyses

Compared with HC, the CHR group demonstrated: Significantly exhibited further deterioration in processing speed (higher scores on TMT-A);(OR=1.08,*p*=0.04). Impaired performance across multiple cognitive domains including: verbal memory [Lower total scores on the HVLT-R;(OR=0.69,*p*<0.001)] and executive function [reduced Stroop Word Test performance (OR=0.93,*p*=0.03)]. Compared with FDR, the CHR group exhibited further deterioration in processing speed [lower DST scores (OR=0.95, p=0.03)], verbal learning [lower total scores on the HVLT-R;(OR=0.69,*p*<0.001)] and visual learning (Lower total scores on the BVMT-R;(OR=1.10,*p*=0.01).

Notably, when comparing first-episode psychosis (FEP) patients with CHR individuals, the FEP group exhibited further deterioration in processing speed as evidenced by significantly lower DST scores (OR=0.94, *p*=0.03). No significant differences in other cognitive measures between these clinical groups (all *p* > 0.05). (Complete statistical parameters are presented in **Table 5**).

**Table 5:** Pairwise comparison of cognitive function between FEP group, CHR group, FDR group and HC group based on binary logistic regression.

	CHR vs. HC ^a^			CHR vs. FDR ^b^			FEP vs. CHR ^c^		
	*P*	OR	95%C.I.	*P*	OR	95%C.I.	*P*	OR	95%C.I.
TMT-A	**0.04**	1.08	1.00~1.17	0.84	0.99	0.95~1.04	0.22	1.02	0.99~1.05
DST	0.24	0.96	0.89~1.03	**0.04**	0.95	0.89~0.99	**0.03**	0.94	0.90~0.99
HVLT-R total	**<0.001**	0.69	0.59~0.82	**<0.001**	0.81	0.72~0.91	0.06	1.09	0.99~1.20
BVMT-R total	0.49	0.97	0.87~1.07	**0.01**	1.10	1.02~1.18	0.68	0.98	0.93~1.05
Stroop words	**0.03**	0.93	0.87~0.99	0.18	0.97	0.93~1.01	0.31	1.02	0.98~1.06
Stroop color	0.77	1.01	0.93~1.11	0.12	0.95	0.90~1.01	0.34	1.03	0.97~1.09
Stroop color words	0.29	1.06	0.95~1.19	0.10	1.07	0.99~1.17	0.07	0.93	0.87~1.01
CPT average score	0.71	0.85	0.35~2.03	0.63	0.86	0.46~1.60	0.54	0.83	0.45~1.52

Bolded *p*-value: *p* < 0.05; OR, odds ratio; 95% C.I., 95% confidence interval; ^a^HC group as reference group, likelihood ratio: 75.82, R2 = 0.76; ^b^FDR group as reference group, likelihood ratio: 129.94, R2 = 0.43; ^c^CHR group as reference group, likelihood ratio: 82.52, R2 = 0.82; Using the ENTER method; BVMT-R, Brief Visuospatial Memory Test Revised; CHR, clinical high risk for psychiatry; CPT, Continuous Performance Test; DST, Digit Symbol Coding Test; FDR, first-degree relatives of psychosis; FEP, first-episode schizophrenia; HC, healthy control; HVLT-R, Hopkins Verbal Learning Test-Revised; TMT-A, Trail Making Test A.

## 4 Discussion

Schizophrenia progression occurs through multiple stages. To elucidate the cognitive traits associated with schizophrenia, this study examined the cognitive functions of patients across five distinct degrees of mental disease risk (HC, FDR, CHR, FEP and MECS). This research is among the few that directly compare the cognitive functions of various groups throughout the continuous spectrum of mental disorders. Additionally, it is one of few studies involving FEP and MECS patients, as well as direct comparisons of the cognitive functions of two high-risk groups (CHR and FDR). The study has four main contributions. First, the MECS, FEP, CHR, and FDR groups demonstrated significantly inferior cognitive function compared to the HC group. Second, the MECS group exhibited the most pronounced cognitive decline among the five groups, with a continuous exacerbation in verbal learning (HVLT-R) and executive function (SCWT) in the FEP group. Third, the FDR and CHR groups also displayed cognitive impairments, with the cognitive decline level of the CHR group falling between those of the FEP and FDR groups. Fourth, the DST emerged as one of the most sensitive cognitive tests for distinguishing the FEP group from the HC group. Furthermore, the HVLT-R proved to be the most sensitive cognitive test in distinguishing the CHR group from both the HC and FDR groups.

In comparison to the HC group, the FEP group exhibited medium to severe cognitive deficits across all individual cognitive tests. This finding aligns with previous research indicating cognitive impairment in FEP patients, even without medication, thereby eliminating potential confounding effects of drug treatment or adverse reactions (29). These results suggest that cognitive impairment is a common symptom in FEP patients. Longitudinal studies on cognitive functioning in first-episode psychosis (FEP) patients suggest relative stability in cognitive profiles over years following illness onset. For instance, only 10% of FEP patients exhibited significant cognitive deterioration or improvement more than two years post-onset (2). However, other research indicates that chronic schizophrenia patients demonstrate poorer performance in specific cognitive domains compared to FEP individuals. For example, Rek-Owodzi´n (19) et al. identified worse working memory in chronic schizophrenia relative to FEP cohorts, while a 2024 meta-analysis corroborated significantly lower executive function in chronic patients—a pattern consistent with our findings (30). In this study, the multi-episode chronic schizophrenia (MECS) group showed inferior performance in verbal learning (HVLT-R) and executive function (SCWT) compared to the FEP group. This suggests that cognitive functioning, particularly verbal learning and processing speed, further deteriorates as the disease progresses. These findings underscore the importance of addressing cognitive functioning at the FEP stage, especially in verbal learning and executive function domains. Future research could benefit from longitudinal follow-up studies to examine the potential impact of cognitive functioning on relapse rates in patients with schizophrenia.

In this study, the CHR group exhibited cognitive deficits across all tests, with most deficits falling between those observed in the FEP and FDR groups. *Post hoc* analyses revealed similarities between the CHR and FEP groups in several areas, including verbal learning (HVLT-R), executive functioning (SWCT), and visual learning (BVMT-R), aligning with previous research findings. Notably, patients with CHR syndrome demonstrated significant cognitive impairment prior to the full manifestation of psychiatric disorders (11, 31). A meta-analysis further corroborated these findings, indicating that CHR patients experience impairments in general intelligence and across all cognitive subdomains (32).

In comparison to HC, first-degree relatives (FDRs) of FEP patients, including unaffected siblings, parents, and offspring, exhibited significant cognitive decline. However, they outperformed the CHR group and the FEP group in most cognitive aspects. The presence of pre disease cognitive deficits supports the neurodevelopmental model of schizophrenia, which conceptualizes schizophrenia-related cognitive impairment as neurodevelopmental abnormalities occurring in the first two decades of life. This model suggests a deficit in cognitive function acquisition compared to healthy individuals. The manifestation of cognitive deficits several years prior to the emergence of psychiatric symptoms indicates that cognitive impairment is central to schizophrenia. Neurodevelopmental anomalies are evident as performance delays at school age (33). Kahn and Keefe (34) posited that schizophrenia is primarily a cognitive disorder characterized by neurodevelopmental traits, including cognitive deficits before the onset of mental illness, genetic influence on cognitive function, and cognition's ability to independently predict functional outcomes. Furthermore, a study found that 98% of schizophrenia patients demonstrated cognitive abilities below the levels anticipated based on their mothers' educational attainment, despite significant symptom heterogeneity among these individuals (35).

In the current study, FDR group demonstrated superior performance compared to CHR individuals across most cognitive assessments, a finding that diverges from previous research (17). Prior studies have indicated that both CHR and FDR groups exhibit persistent cognitive deficits (36). The discrepancy in our results may be attributed to differences in sampling methodology. Notably, the CHR and FDR participants in this study were predominantly from the same families, potentially increasing sample homogeneity. It is important to consider that CHR individuals face both genetic and clinical risk factors.

The FEP group demonstrated the most significant effects on the DST score compared to the HC group across several cognitive tests, which is consistent with previous research. Studies have indicated that the DST is the most sensitive cognitive test for differentiating diagnosed patients with schizophrenia or FEP from HCs (37, 38). Digit Symbol Coding tasks, which require participants to substitute characters with correct numbers under time constraints, are considered a measure of processing speed. Processing speed has been identified as a crucial cognitive function influencing various higher-order cognitive operations and may represent the core cognitive deficit in patients with schizophrenia. Previous research has demonstrated that impairments in processing speed are linked to negative functional outcomes for individuals with schizophrenia and CHR (39, 40). In this study, the DST scores of the CHR and FDR groups were proportional to their functions, suggesting that higher DST scores indicate better functioning. However, research suggests that multiple cognitive functions contribute to numerical DST performance, and its overall performance may depend more on executive function than on the cognitive components of the processing speed domain. Future studies will explore the cognitive elements of the numerical DST to determine whether a processing speed deficit can effectively differentiate the FEP group from the HC group.

Moreover, among the cognitive assessments administered to the CHR and HC groups, the HVLT-R demonstrated the most significant effect, a finding further supported by logistic regression analysis. This observation aligns with previous research, suggesting that verbal learning assessments are among the most sensitive cognitive evaluations for distinguishing between CHR and HC groups. The HVLT-R assesses verbal learning ability by evaluating short-term memory, delayed memory, and recognition capacity, thereby representing working memory. Prior studies have demonstrated that the HVLT-R is a prominent indicator of cognitive impairment in individuals with schizophrenia (40, 41). Additionally, the HVLT-R has been shown to be the most effective cognitive test for differentiating CHR and FDR individuals. This study proposes that evaluating verbal learning through the HVLT-R may enhance the identification of the CHR state, as it may be more closely associated with status markers.

This study offers several notable strengths: (1) It incorporates patients with varying risk levels of mental disorders from the same geographical area. (2) It employs standardized cognitive assessment tools to evaluate and compare cognitive functioning across different groups. (3) It eliminates the potential confounding effect of medication on cognitive performance, as neither the Clinical High Risk (CHR) nor the First-Degree Relative (FDR) group received pharmacological interventions, thus enhancing the reliability of the findings.

This study presents several limitations despite its aforementioned advantages: (1) The cross-sectional design fundamentally limits our capacity to determine whether baseline cognitive deficits predict subsequent transition to schizophrenia in CHR individuals—a critical gap given the clinical imperative to identify reliable prognostic biomarkers. Longitudinal tracking is indispensable not only to quantify conversion rates and validate cognitive predictors, but crucially to assess whether early cognitive remediation can decelerate or alter pathological trajectories. (2) While various scales have been reported and applied for assessing cognitive functions, the scales utilized in this study may not comprehensively cover all aspects of cognitive function. (3) the FEP and MECS groups, nor did it account for premorbid IQ or socioeconomic status (SES)—factors known to influence cognitive performance in schizophrenia-spectrum populations. While these unmeasured variables may partially confound observed neurocognitive differences, the current findings should be interpreted as preliminary reflections of disease-associated cognitive patterns. Future investigations would benefit from incorporating pharmacological monitoring and sociocognitive covariates to refine phenotypic characterization. (4) Significant sample size differences in key subgroups (e.g., n = 94 in the FEP group vs. n = 40 in the MEC group) may result in limited statistical power, and effect size estimates in small subgroups may be biased and need to be validated in larger samples.

## 5 Conclusions

MECS Patients experience the most severe cognitive deficits, with progressive deterioration in verbal learning and executive function. CHR and FDR groups also exhibit cognitive impairments, with CHR individuals demonstrating a level of cognitive decline intermediate between FEP patients and FDR individuals. Among the various cognitive tests administered, the FEP group showed the most significant cognitive impairment (compared to the healthy control (HC) group) in the Digit Symbol Coding Test (DST). Additionally, the Hopkins Verbal Learning Test-Revised (HVLT-R) proved to be the most effective cognitive assessment for distinguishing the CHR group from both the HC and FDR groups. Future research should focus on longitudinal studies to elucidate the role of cognitive functioning across different stages of schizophrenia. Such studies may contribute to improving the prognosis of schizophrenia by facilitating the development of targeted cognitive training interventions for individuals at various stages of the disorder.

## Data Availability

The raw data supporting the conclusions of this article will be made available by the authors, without undue reservation.
